# 3D-printed holders for *in meso in situ* fixed-target serial X-ray crystallography

**DOI:** 10.1107/S1600576720002897

**Published:** 2020-04-23

**Authors:** Chia-Ying Huang, Nathalie Meier, Martin Caffrey, Meitian Wang, Vincent Olieric

**Affiliations:** a Paul Scherrer Institute, Forschungsstrasse 111, Villigen-PSI, 5232, Switzerland; b Membrane Structural and Functional Biology Group, Trinity Biomedical Sciences Institute, Trinity College, Dublin 2, D02 R590, Ireland

**Keywords:** 3D-printed holders, IMISX, *in meso in situ* serial X-ray crystallography, fixed targets, post-crystallization treatments

## Abstract

The *in meso in situ* serial X-ray crystallography method was developed to ease the handling of small fragile crystals of membrane proteins and for rapid data collection on hundreds of microcrystals directly in the growth medium without the need for crystal harvesting. To facilitate mounting of these *in situ* samples on a goniometer at cryogenic or at room temperatures, two new 3D-printed holders have been developed. They provide for cubic and sponge phase sample stability in the X-ray beam and are compatible with sample-changing robots. The holders can accommodate a variety of window material types, as well as bespoke samples for diffraction screening and data collection at conventional macromolecular crystallography beamlines. They can be used for convenient post-crystallization treatments such as ligand and heavy-atom soaking. The design, assembly and application of the holders for *in situ* serial crystallography are described. Files for making the holders using a 3D printer are included as supporting information.

## Abstract 2

The design and assembly of two 3D-printed holders for high-throughput *in meso* *in situ* fixed-target crystallographic data collection are described.

## 1. Introduction   

**Figure 1:**
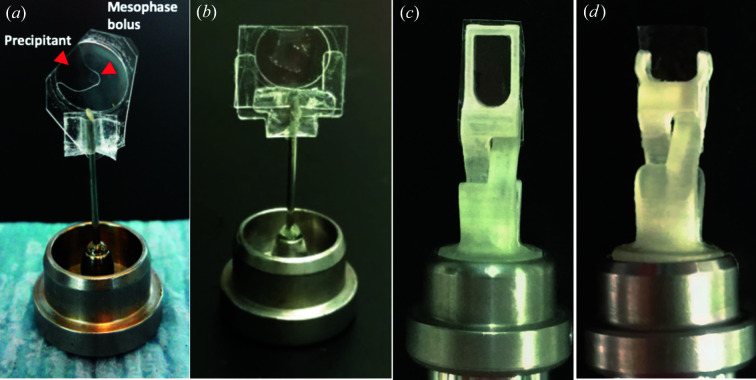
Past and present IMISX sample holders. (*a*) The first holder of its kind. It imposed slight curvature in the sachet to provide mechanical stability. The sachet was affixed to a mounting pin on a magnetic base. Adapted under a Creative Commons Attribution (CC-BY) License (https://creativecommons.org/licenses/by/2.0) from Huang *et al.* (2016 ▸). (*b*) The Y-shaped support. In many ways, it is analogous to the holder shown in (*a*) with the exception that it has a flat as opposed to a curved sachet and sample. In this holder, mechanical stability comes from the uprights in the Y-shaped support. (*c*), (*d*) The 3D-printed h1 and h2 holders introduced here.

*In meso* or lipid cubic phase crystallization has played an important role in X-ray structure determination of membrane proteins, especially G-protein coupled receptors and the complexes they form (Cherezov *et al.*, 2007 ▸; Jaakola *et al.*, 2008 ▸; Chien *et al.*, 2010 ▸; Wu *et al.*, 2010 ▸; Caffrey, 2015 ▸). The cubic phase has also found application as a native-like medium in which to port crystals across beams for time-resolved serial crystallography at synchrotron (Nogly *et al.*, 2015 ▸; Weinert *et al.*, 2017 ▸) and free-electron laser X-ray sources (Weierstall *et al.*, 2014 ▸; White *et al.*, 2016 ▸; Nogly *et al.*, 2018 ▸; Cheng, 2020 ▸). The recently developed *in situ in meso* serial X-ray crystallography (IMISX) method uses perforated double-stick tape to create wells for crystallization and supporting sachets (IMISX wells) in which to deliver the crystal-laden mesophase to the X-ray beam without the need for direct crystal harvesting (Huang *et al.*, 2015 ▸, 2016 ▸, 2018 ▸; El Ghachi *et al.*, 2018 ▸; Apel *et al.*, 2019 ▸; Cheng *et al.*, 2019 ▸). The original IMISX method involved securing a slightly curved thin-film sachet to a pin on a goniometer (Fig. 1 ▸) (Huang *et al.*, 2016 ▸). Curvature was imposed to provide mechanical stability to the sachet in the cryostream. A Y-shaped support was introduced subsequently to facilitate data collection over a large flat – as opposed to a curved – sample area. However, this holder proved to be time consuming to assemble and not particularly practical to use (Huang *et al.*, 2018 ▸). Separately, self-closing clips were developed to improve the efficiency of *in situ* sample preparation. These included the DiffraX metal sample holder (Axford *et al.*, 2016 ▸) and a 3D-printed device (Broecker *et al.*, 2016 ▸, 2018 ▸). Here we introduce two new 3D-printed sample holders. Compared with previous clips, they are extremely easy to assemble – from two identical pieces. They provide increased mechanical stability, they limit ice formation when collecting diffraction data in a cryogenic stream and they facilitate post-crystallization treatments. The first holder, h1, is suited to samples in which crystals have been grown and remain in the viscous mesophase. The second, h2, was designed for samples in which the more fluid variant of the cubic phase, the sponge phase (Cherezov *et al.*, 2006 ▸; Caffrey, 2015 ▸), has formed. Both holders can be used for room-temperature (RT, 293 K) data collection and are compatible with sample-changing robots. They provide for a wide scanning area and are suitable for post-crystallization treatments such as ligand and heavy-atom soaking (Li *et al.*, 2015 ▸; Rucktooa *et al.*, 2018 ▸; Huang *et al.*, 2018 ▸). The holders can be used with a host of *in situ* window materials such as cyclic olefin polymer (COP) (Axford *et al.*, 2016 ▸; Apel *et al.*, 2019 ▸), cyclic olefin copolymer (COC) (Huang *et al.*, 2015 ▸, 2016 ▸; El Ghachi *et al.*, 2018 ▸), biaxially oriented polyethylene tere­phthal­ate (Mylar) (Broecker *et al.*, 2016 ▸) and silicon nitride (Cherezov & Caffrey, 2007 ▸; Murray *et al.*, 2015 ▸; Roedig *et al.*, 2015 ▸; Owen *et al.*, 2017 ▸). Furthermore, the holders are 3D printed and reusable.

## 2. Design and use of 3D-printed IMISX sample holders   

**Figure 2:**
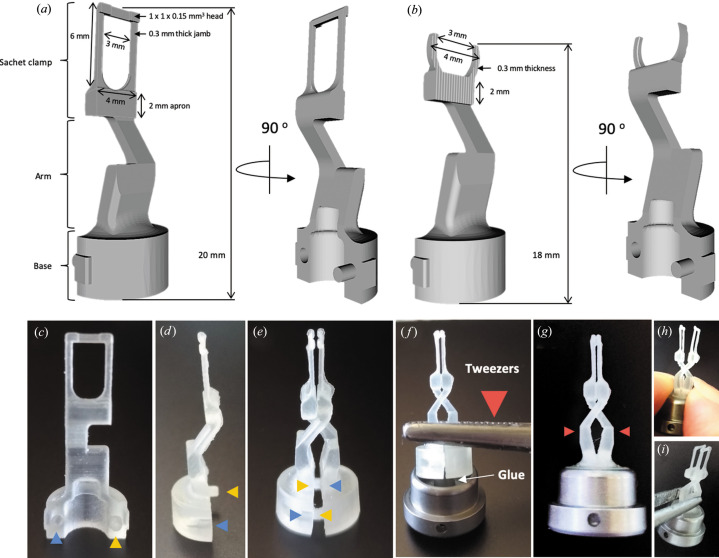
3D-printed IMISX holders and assembly for goniometer-based X-ray diffraction data collection. (*a*), (*b*) Technical drawings of the h1 and h2 IMISX holders. Orthogonal views of one half of a holder are shown. Front (*c*) and side (*d*) views of one half of an h1 holder before assembly. The yellow and blue arrowheads mark the location of the male and female alignment parts at the base of the holder. (*e*) A complete holder consists of two halves. It is assembled by intertwining the clamps and arms and slotting into place the complementary male and female parts at the base of the holder. (*f*) The assembled holder is mounted in a goniometer magnetic base using tweezers and is held in place with fast-acting glue. (*g*) The opposing jaws of the clamp can be opened or closed by pressing on or releasing the flexible criss-crossing arms (red arrowheads) with (*h*) a finger and thumb or (*i*) tweezers.

The IMISX sample holders are made of poly(methyl methacrylate) (PMMA), also known as acrylic or plexiglass, a plastic material with exceptional strength and rigidity [Figs. 2 ▸(*a*) and 2 ▸(*b*)]. PMMA proved to be particularly suitable for the faithful printing of fine details (on the scale of 0.1 mm) and for providing a surface with a uniformly smooth finish. Fabrication was done using a 3D printer (ProJet MJP 2500 Plus, 3D Systems, USA). Both holder types have three functional parts: a base, an arm and a sachet clamp (Fig. 2 ▸). In the first holder type, h1, the clamp is rectangular in outline and has a four-sided frame [Fig. 2 ▸(*a*)]. The second type, h2, has a semi-circular clamp with an open end and has a larger sample area than h1 [Fig. 2 ▸(*b*)]. Both holders are built on the principle of reverse or self-closing tweezers. They are assembled from identical halves that perfectly align together via mating male (pin) and female (hole/receptacle) connectors at the base of the holder [Figs. 2 ▸(*c*)–2 ▸(*e*), yellow and blue arrows]. The coupled pair is inserted into a commercial magnetic base (CryoCaps from Mol­ecular Dimension, UK) and secured in place with glue (instant glue, ethyl 2-cyanoacrylate, Cementit, Switzerland) [Figs. 2 ▸(*f*)–2 ▸(*g*)]. The holder can be opened and closed for loading and unloading simply by pressing on and releasing the criss-crossed arms of the device using a finger and thumb [Fig. 2 ▸(*h*)] or tweezers [Fig. 2 ▸(*i*)] (Axford *et al.*, 2016 ▸; Broecker *et al.*, 2018 ▸).

**Figure 3:**
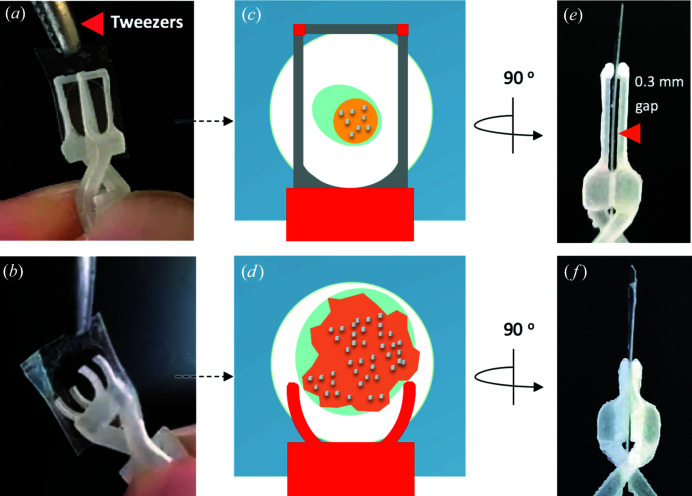
Securing the IMISX sachet in the holder clamp. (*a*), (*b*) A sachet containing a crystal-laden mesophase bolus removed from an IMISX crystallization plate is placed between and secured by the jaws of the clamp. (*c*), (*d*) Cartoon representation of the sachet in the clamp of an h1 and an h2 holder. The cubic phase bolus, the sponge phase bolus, the precipitant solution and crystals are shown in yellow, orange, blue and gray, respectively. (*e*), (*f*) Side view of the IMISX sachet secured in place between the jaws of the clamps in the h1 and h2 holders. The gap between the jaws of the clamp, marked with an arrowhead in (*e*), facilitates soaking of crystals with ligands, heavy atoms and wash solutions.

The sachet clamp in h1 consists of a 4 × 2 × 1.4 mm apron, two 1 × 6 × 0.3 mm jambs and two 1 × 1 × 0.15 mm heads [Fig. 2 ▸(*a*)]. These serve to pinch and, in so doing, to hold the sachet firmly in place, leaving a 0.3 mm gap for post-crystallization treatments [Fig. 3 ▸(*e*)]. The mesophase-laden sachet is prepared by cutting it out of the IMISX plate with outside margins of approximately 2 mm. An online open-access movie is available to show how this is done (Huang *et al.*, 2016 ▸). The freed sachet is then secured in the clamp by adjusting its jaws with the criss-crossed arms by hand or with tweezers [Figs. 3 ▸(*a*) and 2 ▸(*i*)]. To facilitate centering once the sample is mounted on the goniometer, the bolus of mesophase in the sachet is centered in the window of the clamp [Fig. 3 ▸(*c*)]. The clamp in h2 is similar to that in h1 with the exception that it incorporates a semi-circular frame with an outside diameter of 4 mm [Fig. 2 ▸(*b*)]. Positioning a sachet in h2 is done as described for the h1 holder [Figs. 3 ▸(*b*), 3 ▸(*d*) and 3 ▸(*f*)].

The oscillation range that is possible with these holders is ±30°. It is important to pre-align the pin-mounted holders before placing them on the goniometer so that they end up with the plane of the sachet perpendicular to the X-ray beam and, for cryogenic data collection, with an optimal orientation with respect to the cryostream to reduce frosting. The holders can be frozen to liquid-nitrogen temperatures, thawed and reused.

## 3. Post-treatment capabilities of the 3D-printed IMISX sample holders   

**Figure 4:**
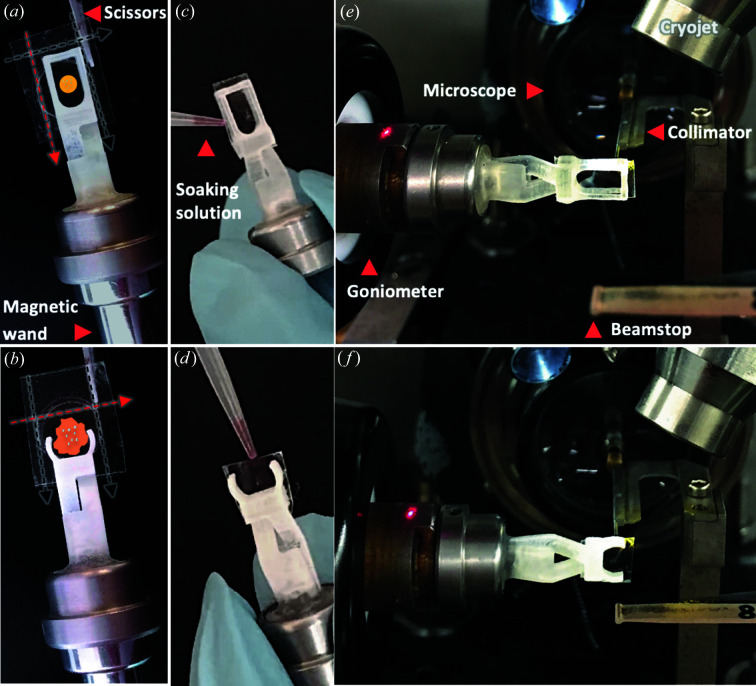
Procedures for soaking crystals in an IMISX sachet in 3D-printed holders. (*a*), (*b*) After the sachet has been mounted in the clamp as indicated in Fig. 3 ▸, the edges of the sachet are trimmed with a pair of scissors along the black dashed lines taking care not to cut across (open) the well. The well is opened by cutting along the dashed red line. The yellow disc in (*a*) and the orange random shape in (*b*) represent viscous mesophase and sponge phase boluses, respectively, in ideal positions in the windows of the clamp for data collection. (*c*), (*d*) Soak solution containing ligands, heavy atoms or wash solution is added to the bolus through the side or top openings in the sachet. (*e*), (*f*) View of the h1 and h2 holders mounted on the goniometer at the SLS beamline X06SA-PXI.

The IMISX sachets greatly facilitate crystal soaking with ligands and heavy atoms (Huang *et al.*, 2018 ▸). The samples are protected at all times during the process by controlling local temperature and humidity. To perform a soaking exercise, the sachet is first opened by cutting it with a pair of scissors along one side of the rectangular h1 clamp [Fig. 4 ▸(*a*)] or across the top of the round sachet in the h2 holders [Fig. 4 ▸(*b*)]. The standoffs in the clamp of the h1 holder provide a 0.3 mm gap between the two frames [Fig. 3 ▸(*e*)] for dispensing soaking and wash solutions [Fig. 4 ▸(*c*)]. In the h2 holder, solutions are easily added to and removed from the exposed cut end of the sachet [Fig. 4 ▸(*d*)]. Prior to data collection at RT or storage in liquid nitrogen, the samples can be kept during the soaking interval (typically less than 1 h) at 293 K in a closed box with a wet tissue to minimize dehydration.

## 4. Data collection with the 3D-printed IMISX sample holders   

To assess the quality of data that can be collected with both h1 and h2 holders at ambient and cryogenic temperatures, we used crystals of native lysozyme (Lyso native RT), bromine-derivatized lysozyme (LysoBr cryo and LysoBr RT) and the membrane protein PepT_St_ (PepT_St_ cryo) (Supplementary Table S1). With lysozyme, an IMISX sachet with *in meso*-grown crystals (10 × 20 × 30 µm) was removed from an IMISX plate, mounted on an h1 holder and soaked with sodium bromide (see supporting information) as described previously (Fig. 4 ▸). The sample was mounted using the TELL sample changer (Martiel *et al.*, 2020 ▸) and measured at 100 K using a wavelength of 0.91969 Å at the Swiss Light Source (SLS) beamline X10SA-PXII with a beam of 20 × 10 µm and flux values of 5 × 10^11^ photons s^−1^. The data sets collected from 12 crystals over 20° wedges for each crystal were merged and used for phasing by Br single-wavelength anomalous diffraction with *SHELXC*/*D* (Sheldrick, 2010 ▸) and *Crank2* (Skubák & Pannu, 2013 ▸) [Supplementary Table S1 and Supplementary Figs. 1(*a*) and 1(*c*)]. The structure was refined to *R*_work_/*R*_free_ values of 0.19/0.21 at a resolution of 1.80 Å.

In the case of PepT_St_ cryo, crystals grow in the sponge phase. Accordingly, measurements were made with an h2 holder. The crystals, with maximum dimensions of 10 × 15 × 15 µm, were used for data collection at a wavelength of 1 Å on SLS beamline X06SA-PXI at 100 K with a beam of 20 × 10 µm and flux values of 3.7 × 10^11^ photons s^−1^. Twenty-nine data sets over 10° wedges for each crystal were combined to obtain complete data to a resolution of 2.53 Å (Supplementary Table S1). The structure was solved by molecular replacement using PDB entry 5d58 (Huang *et al.*, 2016 ▸) as the model and refined to *R*_work_/*R*_free_ values of 0.23/0.26.

The h1 holder has also been tested with data collection at RT using the same lysozyme native crystals (Lyso native RT) and LysoBr crystals (LysoBr RT) described above at a wavelength of 0.91881 Å on SLS beamline X06SA-PXI at 293 K with a beam of 20 × 10 µm and flux values of 1.4 × 10^10^ photons s^−1^. The Br anomalous signal from 200 LysoBr data sets at RT over 10° wedges for each crystal was combined with high-resolution data from 15 Lyso native RT crystals over 10° wedges for each crystal for phasing with single isomorphous replacement with anomalous scattering using *SHELXC*/*D* and *Crank2* [Supplementary Table S1 and Supplementary Figs. 1(*b*) and 1(*d*)]. The structure was refined to *R*_work_/*R*_free_ values of 0.18/0.21 using Lyso native RT at a resolution of 1.8 Å.

## 5. Discussion and remarks   

The development of serial crystallographic data collection methods is a perpetual pursuit at synchrotron and XFEL facilities worldwide. The sample holders described here were designed to meet the need for convenient *in meso* sample preparation and high-throughput *in situ* crystallographic data collection. The new holders are easy to assemble and load with sachets cut from IMISX plates and lend themselves to convenient ligand and heavy-atom soaking and screening of crystals *in situ* in a protective environment. The holders can be used to store samples under cryogenic conditions for subsequent high-throughput screening with automated sample changers. High-quality data can be obtained at 100 K courtesy of the mechanical stability of the IMISX sachet in the cryogenic stream when mounted in the new 3D-printed holders. In addition, the low profile of the holders has the effect of minimally disturbing the cryogenic gas stream flow, thereby reducing ice formation on the sample. The holders can also be used for data collection at ambient temperatures. They are compatible with a variety of window material types including COC, COP, Mylar and silicon nitride and can be used for serial crystallographic measurements at home, synchrotron and free-electron laser X-ray sources.

The IMISX method was developed to enable the handling of fragile crystals in a viscous mesophase and for highly efficient, close-to-automatic crystallographic data collection at ambient and cryogenic temperatures. The new holders introduced here make sample preparation, handing and post-crystallization treatment (ligand and heavy-atom screening) extremely easy. With access to a 3D printer, the new holders can be generated quickly and inexpensively using the open-access files associated with this paper. Furthermore, refinements can be made to suit specific applications by simply modifying the files and/or by using different fabricating materials. We believe these holders will prove to be generally useful and will contribute to expediting the structure determination of proteins and complexes that are scientifically and medically important.

## 6. Related literature   

The following additional literature is cited in the supporting information: Basu *et al.* (2019 ▸), Kabsch, W. (2010*a* ▸,*b* ▸), Lyons *et al.* (2014 ▸), McCoy *et al.* (2007 ▸) and Pape & Schneider (2004 ▸).

## 7. 3D-printer files   

Files for the sample holders are available in .stl format as supporting information.

## Supplementary Material

3D-printer file: h1 holder. DOI: 10.1107/S1600576720002897/gj5239sup1.stl

3D-printer file: h2 holder. DOI: 10.1107/S1600576720002897/gj5239sup2.stl

Supplementary Information. DOI: 10.1107/S1600576720002897/gj5239sup3.pdf
