# Supplementary material for: 3D-printed holders for in meso in situ fixed-target serial X-ray crystallography
Source: J Appl Crystallogr. 2020 Apr 23;53(Pt 3):854–9. doi: 10.1107/S1600576720002897 (PMC7312129; doi:10.1107/S1600576720002897)
Supplement: Supplementary file 3 [file j-53-00854-sup3.pdf]

Supplementary Information

3D-printed holders for *in meso in situ* fixed-target serial X-ray crystallography

Chia-Ying Huang[a*], Nathalie Meier[a], Martin Caffrey[b], Meitian Wang[a], Vincent Oliéric[a]

[a]Paul Scherrer Institute, Forschungsstrasse 111, Villigen-PSI, 5232, Switzerland

[b]Membrane Structural and Functional Biology Group, Trinity Biomedical Sciences Institute, Trinity College, Dublin 2, Ireland.

* Correspondence and requests for materials should be addressed to Chia-Ying Huang (email: chia-ying.huang@psi.ch)

Protein, crystallization and sample preparation:

Lysozyme was sourced from Sigma (St. Louis, MO), and $PepT_{St}$ was produced recombinantly in *Escherichia coli* and purified from biomass following published protocols (Lyons *et al.*, 2014). Crystals of lysozyme and $PepT_{St}$ were prepared using the LCP method in IMISX plates as described previously (Huang *et al.*, 2015). Br-derivatized lysozyme crystals (LysoBr) were obtain by soaking the native lysozyme crystal with a buffer consisting of 0.7 M NaBr, 50–100 mM sodium acetate pH 4.5, 15–30%(v/v) PEG 400 for 60 min at 20 °C using the IMISX-soaking method describe in the main text. The lysozyme and $PepT_{St}$ crystal-containing sachets were removed from the IMISX plates and mounted in the h1 and h2 holder, respectively.

Data collection, structural phasing and refinement

Data collection with LysoBr (LysoBr cryo) crystals was performed at 100 K at SLS beamline X10SA-PXII. $PepT_{St}$ cryo, Lyso native RT and LysoBr RT measurements were made at SLS beamline X06SA-PXI, of which $PepT_{St}$ cryo data were collected at 100 K and Lyso native RT and LysoBr RT were collected at 293 K. All crystals were measured by an automated serial data collection protocol (CY+) as described previously (Basu *et al.*, 2019) using the following parameters: 0.1 and 0.03 s exposure time for data collection at 100 K and 293 K, respectively, and 0.1-0.2° oscillation with 10-20° wedge for each crystal. The data were indexed and processed with *XDS* (Kabsch, 2010b, a), and scaled and merged with *XSCALE*. The structure of $PepT_{St}$ cryo was phased by molecular replacement using Phaser (McCoy *et al.*, 2007) with 5D58 as search template. LysoBr cryo was phased using Br-SAD and Lyso RT was phased using Br-SIRAS (Lyso native RT and LysoBr RT in **Supplementary Table 1**). The *HKL2MAP* (Pape & Schneider, 2004) interface of *SHELX C/D/E* (Sheldrick, 2010) and *CRANK2* (Skubak & Pannu, 2013) were used for phasing of both LysoBr cryo and Lyso RT. Figures of molecular structures were generated with PyMOL (http://www.pymol.org). Data collection and refinement statistics are included in **Supplementary Table 1**.

Supplementary Table 1. Data collection and refinement statistics*

	$PepT_{St}$ cryo	LysoBr cryo	LysoBr RT	Lyso native RT
Wavelength (Å)	1.00002	0.91969	0.91881	0.91881
Beamline	SLS X06SA-PXI	SLS X10SA-PXII	SLS X06SA-PXI	SLS X06SA-PXI
Holder type used for data collection	h2	h1	h1	h1
Data collection temperature (K)	100	100	293	293
Space group	$C222_1$	$P4_32_12$	$P4_32_12$	$P4_32_12$
Unit cell (Å, °)	*a* = 101.34; *b* = 108.98; *c* = 111.18 $\alpha = \beta = \gamma = 90$	*a* = 78.29; *b* = 78.29; *c* = 38.25 $\alpha = \beta = \gamma = 90$	*a* = 78.8; *b* = 78.8; *c* = 38.27 $\alpha = \beta = \gamma = 90$	*a* = 78.8; *b* = 78.8; *c* = 38.27 $\alpha = \beta = \gamma = 90$
No. of merged data sets	29	12	200	15
Total Data (°)	290	240	2000	150
Resolution (Å)	48.93-2.53 (2.60-2.53) **	35.02-1.80 (1.85-1.80)	39.40-2.00 (2.20-2.00)	35.31-1.80 (1.85-1.80)
Completeness (%)	99.80 (99.30)	99.90 (99.40)	100 (100)	99.0 (98.5)
Multiplicity	9.42 (5.71)	6.95 (6.44)	76.98 (76.37)	9.89 (4.66)
R_{meas}	0.52 (2.90)	0.14 (2.52)	0.36 (2.26)	0.23 (1.70)
$I / \sigma I$	5.07 (0.75)	9.64 (1.42)	15.67 (2.87)	7.36 (0.75)
$CC_{1/2}$ (%)	99.40 (12.30)	99.60 (29.30)	99.90 (89.80)	99.60 (27.40)
CC_{anom} (%)	-	35	21	-
**Phasing method**	MR	Br-SAD	Br-SIRAS	
SHELXD resolution range (Å)	-	35.02-2.42	39.4-2.30	
SHELXD CC_{all}/CC_{weak} (%)	-	27.50/14.10	18.10/8.20	
Heavy atom sites	-	7 Br	7 Br	
**Refinement*** **				
Resolution (Å)	48.93-2.53	35.01-1.80	-	35.31-1.80
No. of unique reflections	20,783	11,490	-	11,728
R_{work}/R_{free}	0.23/0.26	0.19/0.21	-	0.18/0.21
No. atoms				
Protein	3,467	1000	-	1013
Ligand/ion	463	19	-	19
Water	26	96	-	64

Average B-factor				
Proteins	70.30	28.85	-	31.31
Ligand/ion	82.80	39.13	-	47.64
Water	59.62	33.14	-	39.70
R.m.s. deviations				
Bond lengths (Å)	0.005	0.006	-	0.002
Bond angles (°)	1.06	0.800	-	0.500

* Data processing are reported with Friedel pairs merged for PepT$_{St}$ cryo and Lyso native RT, and Friedel pairs separated for LysoBr cryo and LysoBr RT.
** Values in parentheses are for the highest resolution shell.
*** Refinement statistics are reported with Friedel pairs merged.

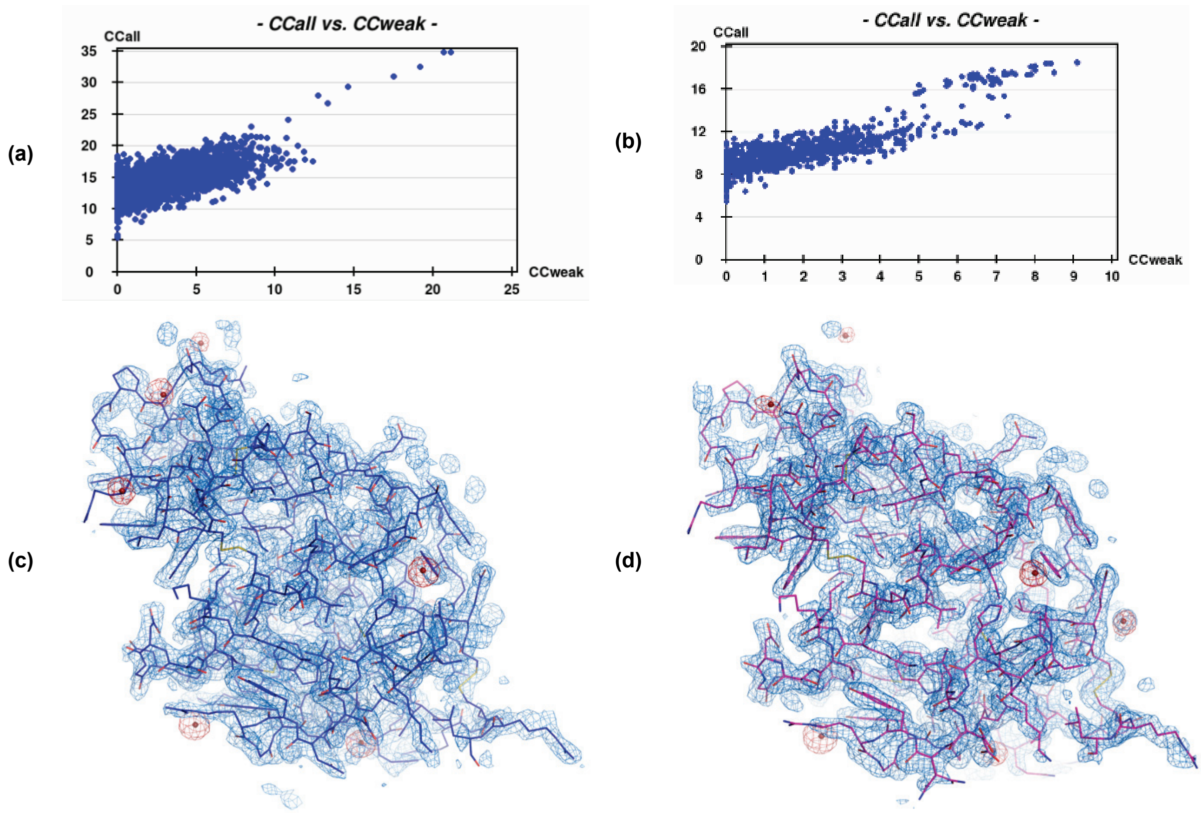

Supplementary Figure 1. Sub-structure determination and phasing of bromine-labelled lysozyme (LysoBr). Correlation coefficients (*CC_{all}/CC_{weak}*) results from *SHELXD* for measurements made with LysoBr at 100 K (a) and at 293 K (b). Experimentally phased electron density map from *CRANK2* for the measurement made with LysoBr at 100 K (c) and at 293 K (d). The anomalous difference map contoured at 4 σ (red mesh) is shown with bromide ions in sphere representation. The experimentally phased electron density maps were contoured at 1 σ (blue mesh) with the final refined model of lysozyme shown in stick representation. The structures of LysoBr cryo and Lyso RT are shown in stick representation in blue (c) and magenta (d), respectively.

Supplementary References

Basu, S., Kaminski, J. W., Panepucci, E., Huang, C. Y., Warshamanage, R., Wang, M. & Wojdyla, J. A. (2019). *J Synchrotron Radiat* **26**, 244-252.

Huang, C. Y., Olieric, V., Ma, P., Panepucci, E., Diederichs, K., Wang, M. & Caffrey, M. (2015). *Acta Crystallogr D Biol Crystallogr* **71**, 1238-1256.

Kabsch, W. (2010a). *Acta Crystallogr D Biol Crystallogr* **66**, 125-132.

Kabsch, W. (2010b). *Acta Crystallogr D Biol Crystallogr* **66**, 133-144.

Lyons, J. A., Parker, J. L., Solcan, N., Brinth, A., Li, D., Shah, S. T., Caffrey, M. & Newstead, S. (2014). *EMBO Rep* **15**, 886-893.

McCoy, A. J., Grosse-Kunstleve, R. W., Adams, P. D., Winn, M. D., Storoni, L. C. & Read, R. J. (2007). *J Appl Crystallogr* **40**, 658-674.

Pape, T. & Schneider, T. R. (2004). *Journal of Applied Crystallography* **37**, 843-844.

Sheldrick, G. M. (2010). *Acta Crystallogr D Biol Crystallogr* **66**, 479-485.

Skubak, P. & Pannu, N. S. (2013). *Nat Commun* **4**, 2777.
